# TOXIC HOMES AND COMMUNITIES? EXPOSURE TO URBAN FIRES AND INCREASED LIFE COURSE DEMENTIA RISK

**DOI:** 10.1093/geroni/igae098.2125

**Published:** 2024-12-31

**Authors:** Jessica Finlay, Grace Bowman, Matthew Miller, Colleen Reid

**Affiliations:** University of Colorado Boulder, Boulder, Colorado, United States; University of Colorado BioFrontiers Institute, Boulder, Colorado, United States; University of Colorado Boulder, Boulder, Colorado, United States; University of Colorado Boulder, Boulder, Colorado, United States

## Abstract

Little is known about the health impacts of wildland urban interface (WUI) fires, which are increasingly common and mostly burn human-made materials. The Marshall Fire Community Health Research Study investigated a destructive WUI fire that affected communities of Superior and Louisville (CO) on December 30, 2021. Semi-structured seated and mobile interviews conducted July through November 2023 probed for perceived impacts on physical, mental, social, and cognitive health. Participants (n=44) were on average 52 years old (±16 years), 86% identified as Non-Hispanic White, 67% female, 86% homeowners, and 46.5% of participant homes were fully destroyed (53.5% smoke damaged). Reflexive thematic analysis identified behavioral and social pathways that may pose longer-term risk for Alzheimer’s Disease and Related Dementias across the life course. This includes early-life educational disruptions (e.g., displacement and stress/anxiety) and mid-life increased risk for hypertension, obesity, and alcohol consumption through heightened stress, lack of time and motivation for diet and exercise, maladaptive coping mechanisms, poor sleep quality, and reduced access to health-promoting services. Later life factors include increased risk for depression, social isolation, physical inactivity, diabetes, and exposure to poorer air quality. These risks varied by individual factors such as education, employment, insurance coverage, type of fire damage, and household composition. Individuals whose homes were smoke damaged may be particularly vulnerable. The study identifies novel behavioral and social pathways through which WUI fires may pose multifaceted and long-term cognitive health risks beyond the acute recovery phase.

